# Hierarchical NiCo_2_O_4_ Hollow Sphere as a Peroxidase Mimetic for Colorimetric Detection of H_2_O_2_ and Glucose

**DOI:** 10.3390/s17010217

**Published:** 2017-01-23

**Authors:** Wei Huang, Tianye Lin, Yang Cao, Xiaoyong Lai, Juan Peng, Jinchun Tu

**Affiliations:** 1 State Key Laboratory of Marine Resource Utilization in South China Sea, Key Laboratory of Tropical Biological Resources of Ministry of Education Hainan University, College of Material and Chemical Engineering, Haikou 570228, China; hw_hnu@aliyun.com (W.H.); HDxuchufeng@163.com (T.L.); cy507@hainu.edu.cn (Y.C.); 2 Laboratory Cultivation Base of Natural Gas Conversion, School of Chemistry and Chemical Engineering, Ningxia University, Yinchuan 750021, China; xylai@nxu.edu.cn (X.L.); pengjuan@nxu.edu.cn (J.P.)

**Keywords:** NiCo_2_O_4_, hierarchical hollow sphere, peroxidase-like, colorimetric

## Abstract

In this work, the hierarchical NiCo_2_O_4_ hollow sphere synthesized via a “coordinating etching and precipitating” process was demonstrated to exhibit intrinsic peroxidase-like activity. The peroxidase-like activity of NiCo_2_O_4_, NiO, and Co_3_O_4_ hollow spheres were comparatively studied by the catalytic oxidation reaction of 3,3,5,5-tetramethylbenzidine (TMB) in presence of H_2_O_2_, and a superior peroxidase-like activity of NiCo_2_O_4_ was confirmed by stronger absorbance at 652 nm. Furthermore, the proposed sensing platform showed commendable response to H_2_O_2_ with a linear range from 10 μM to 400 μM, and a detection limit of 0.21 μM. Cooperated with GOx, the developed novel colorimetric and visual glucose-sensing platform exhibited high selectivity, favorable reproducibility, satisfactory applicability, wide linear range (from 0.1 mM to 4.5 mM), and a low detection limit of 5.31 μM. In addition, the concentration-dependent color change would offer a better and handier way for detection of H_2_O_2_ and glucose by naked eye.

## 1. Introduction

Natural enzymes have been widely studied and applied in various fields due to their high substrate specificity and brilliant catalytic activity under mild conditions [1]. However, application of these enzymes is restricted by their high cost, complicated purification and immobilization procedures, and harsh reaction conditions [2,3]. Therefore, the low-cost enzyme mimics with good stability, excellent catalytic activity, and satisfactory selectivity were highly in demand [4]. Since Gao’s [5] first report on an intrinsic peroxidase-like activity of Fe_3_O_4_ nanoparticles, various nanomaterials such as MFe_2_O_4_ [6,7], MoS_2_ [8], Co_3_O_4_ [9,10], NiO [11], CuS [12], graphene oxide [13], and graphene oxide–Fe_3_O_4_ composite [14] have been further investigated as peroxidase mimetics for H_2_O_2_ detection. These researches reveal that the nanomaterial-based peroxidase mimetics possess the advantages of high specific surface area, controllable morphologies and sizes, and tunable catalytic properties that would be promising candidates for bioassays and clinical diagnosis [4,15,16].

NiCo_2_O_4_ is a typical spinal metal oxide, with Ni ions occupying the octahedral sites while Co ions are integrally distributed to both the octahedral and tetrahedral sites [17,18]. Due to its superior catalytic activity and conductivity to that of single Co_3_O_4_ or NiO [19,20], NiCo_2_O_4_ has gained great attention in the areas of supercapacitor, electrocatalyst, oxidation evolution reaction, and biosensor [21,22]. It has been reported that Co_3_O_4_ nanoparticles possess an intrinsic peroxidase-like and catalase-like activity, and NiO exhibits peroxidase-like activity as well [9,11,23]. The surface of NiCo_2_O_4_ is rich in oxidation states Ni^2+^/Ni^3+^ and Co^2+^/Co^3+^, which make it a better electron transfer mediator for peroxidase substrates (such as 3,3,5,5-tetramethylbenzidine (TMB)) and H_2_O_2_ analysis. Moreover, the superior conductivity of NiCo_2_O_4_ is conducive to improving the electron transfer kinetics within the materials. To the best of our knowledge, the study of NiCo_2_O_4_ as an enzyme mimetic remains unexplored, which greatly inspires the research interest in applying nanostructured NiCo_2_O_4_ to colorimetric detection of H_2_O_2_. Besides, it is well known that the catalytic properties of active materials seriously depend on their morphology and size, which significantly influence the surface efficiency and stability of catalysts [24,25]. The hierarchical hollow structure assembled from particle units is endowed with the advantages of high surface-to-volume ratio, ample mass diffusion pathways, and satisfactory structure stability [26,27]. Therefore, the hierarchical hollow structured NiCo_2_O_4_ materials are expected to function as highly efficient peroxidase mimetics for colorimetric detection of H_2_O_2_ and glucose.

In this paper, the hierarchical NiCo_2_O_4_ hollow sphere was prepared through a facile Cu_2_O-templated approach based on the “coordinating etching and precipitating” (CEP) process, and its peroxidase-like activity was investigated for the first time. Moreover, the peroxidase-like activity of the hierarchical NiCo_2_O_4_ hollow sphere was compared to that of single NiO and Co_3_O_4_ with the presence of TMB and H_2_O_2_ under parallel condition. In addition, the hierarchical NiCo_2_O_4_ hollow sphere was applied to the colorimetric detection of H_2_O_2_. Combined with glucose oxidase (GOx), a novel colorimetric and visual glucose-sensing platform was developed. Finally, the selectivity, reproducibility, and applicability of this proposed sensing platform were assessed by a standard method.

## 2. Experimental

### 2.1. Chemicals

CuCl_2_·2H_2_O, sodium dodecyl sulfate (SDS), NaOH, NH_2_OH·HCl, CoCl_2_·6H_2_O, Na_2_S_2_O_3_·5H_2_O, glucose, and absolute ethanol were purchased from Shanghai Sinopharm Chemical Reagent Co., Ltd. (Shanghai, China). Polyvinylpyrrolidone (PVP, K30, MW ≈ 3800) was provided by Aldrich (Shanghai, China). Citric acid monohydrate, trisodium citrate dihydrate, and 30% H_2_O_2_ were supplied by Guangzhou Chemical Reagent Factory ich (Guangzhou, China). TMB was obtained from Macklin ich (Shanghai, China). Glucose oxidase (GOx, 50 KU) was procured from Sangon Biotech (Shanghai, China) Co., Ltd. (Shanghai, China) and stored in a freezer at −20 °C. All chemical reagents were of analytical grade and were used without further purification.

### 2.2. Apparatus

X-ray diffraction (XRD) analysis was performed using a D8 Tools XRD instrument (Bruker, Karlsruhe, Germany) with a voltage of 40 kV and a current of 30 mA with Cu Kα radiation (*k* = 1.5406 Å). Field-emission scanning electron microscope (SEM) images were obtained by Hitachi S-4800. Transmission electron microscopy (TEM) micrographs were acquired using a JEOL-2100F microscope. The ζ-potential was measured using dynamic light scattering (DLS) at 25 °C (Malvern Nanosizer ZS90). X-ray photoelectron spectroscopy (XPS) data were characterized by Thermo Scientific ESCALAB250 (Thermo Scientific, Waltham, MA, USA) using Al radiation. Nitrogen sorption experiments were carried out at 77 K on a Micro Meritics Tristar II 3020 surface area and pore size analyzer (Norcross, GA, USA). Prior to the measurements, the samples were outgassed at 200 °C in vacuum for 6 h. UV–vis absorption spectra were recorded using a 723PC spectrophotometer (Shanghai Jinghua Science and Technology Co., Ltd., Shanghai, China).

### 2.3. Synthesis of Hierarchical NiCo_2_O_4_ Hollow Sphere

The NiCo_2_O_4_ hollow sphere was synthesized based on a “CEP” process by selecting S_2_O_3_^2−^ as etchant towards the pre-prepared Cu_2_O solid-sphere template. The Cu_2_O solid sphere was obtained according to our previous work [28]. First, 5 mg of the as-prepared Cu_2_O nanospheres, 1.13 mg of CoCl_2_·6H_2_O, 0.57 mg of NiCl_2_·6H_2_O, and 0.33 g of PVP were dissolved into a mixed solution (*V*_ethanol_:*V*_water_ = 1:1, 10 mL), stirred, and sonicated. Then, 4 mL of Na_2_S_2_O_3_ aqueous solution (1 M) was added dropwise into the mixture and kept stirring for about 10 min. The resulting precursor was collected by several rinse–centrifugation cycles with deionized (DI) water and ethanol. Finally, the precursor was calcinated in a muffle furnace at 300 °C for 4 h at a ramp rate of 1 °C·min^−1^ under the air condition.

### 2.4. Catalytic Oxidation of TMB

To investigate the peroxidase-like activity of NiCo_2_O_4_ hollow sphere, catalytic oxidation reaction of TMB in sodium citrate buffer solution was performed in presence of H_2_O_2_. Five microliters of 1 mg·mL^−1^ NiCo_2_O_4_ hollow sphere dispersion was incubated with 3 mL of pre-prepared sodium citrate buffer solution (0.1 M, pH 4.5) in the presence of H_2_O_2_ (0.02 M) and TMB (0.08 mM). The photographs and UV–vis spectra were obtained after incubation at room temperature for 30 min.

### 2.5. Colorimetric Detection of H_2_O_2_

Five microliters of 1 mg/mL NiCo_2_O_4_ hollow sphere dispersion, 25 μL of 10 mM TMB, and 3 μL of H_2_O_2_ in different concentrations were added to 3 mL sodium citrate buffer solution (0.1 M, pH 4.5). The UV–vis absorption spectra were recorded from 500 nm to 800 nm.

### 2.6. Colorimetric Detection of Glucose

Five microliters of 1 mg/mL NiCo_2_O_4_ hollow sphere dispersion, 25 μL of 10 mM TMB, 5 μL of 10 mg/mL GOx, and 3 μL of glucose in different concentrations were added to 3 mL of sodium citrate buffer solution (0.1 M, pH 4.5). The UV–vis absorption spectra were recorded from 500 nm to 800 nm.

## 3. Results and Discussion

### 3.1. Materials Characterization

**Figure 1:**
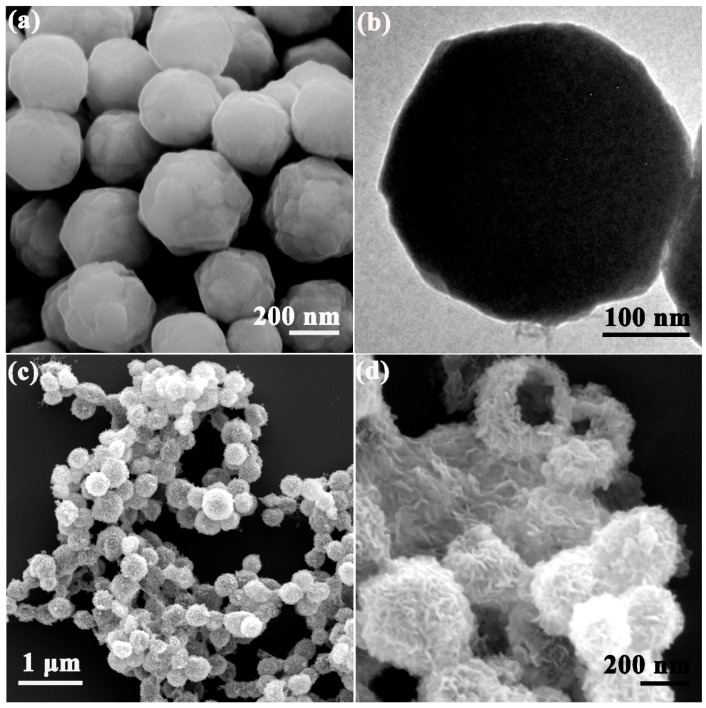
(**a**,**b**) SEM and TEM images of Cu_2_O solid sphere; (**c**,**d**) SEM images of the hierarchical NiCo_2_O_4_ hollow sphere.

The Ni–Co hydroxide precursor was synthesized via a deliberately designed “CEP” process, and the duplication and the elimination of Cu_2_O template were completed simultaneously within 10 min at room temperature [29,30]. The final NiCo_2_O_4_ hollow sphere product was obtained after a simple thermal treatment based on the above processes. As shown in Figure 1a, a high-quality Cu_2_O template with an average size of approximately 400 nm was obtained, and its rough texture on the surface was conducive to creating more chemical etching interface. From the TEM image in Figure 1b, a solid construction can be confirmed. After the etching process and thermal treatment, the obtained NiCo_2_O_4_ sphere (Figure 1c) well inherited the spherical structure of the template, indicating the controllability of the “CEP” process and the stability in structure. The “wormlike” secondary structures can be observed clearly in the magnified image in Figure 1d, and the hollow structure feature can be initially identified by the cracked sphere. Afterwards, we performed TEM characterization to further testify to the inner structure of the hierarchical sphere.

**Figure 2:**
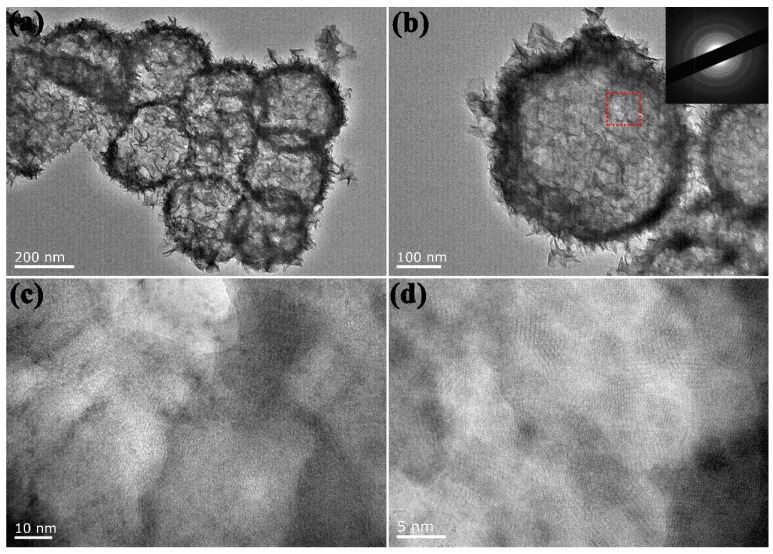
TEM (**a**–**c**) and HRTEM (**d**) images of NiCo_2_O_4_ hollow sphere. Inset in (**b**) is the corresponding selected area electron diffraction (SAED) pattern.

As shown in Figure 2a,b, the distinct contrast between the shells and inner space fully demonstrates the hollow construction of the hierarchical NiCo_2_O_4_ sphere. The diameter of the hollow sphere is about 400 nm, which is similar to that of pre-grown Cu_2_O sphere. Furthermore, the apparent bright and dark aberrations on the ultrathin shell (<30 nm) imply the existence of nanopores, and sheetlike secondary structures of several nanometers in thickness are uniformly grown on the sphere. Notably, the ultrathin hollow sphere with affluent secondary structures could provide more efficient inner and outer surface and create more catalytically active sites. The inserted selected area electron diffraction (SAED) pattern in Figure 2b reveals a polycrystalline feature of the as-prepared material. Figure 2c displays the amplified image of the selected area marked in (b), in which the nanopore and ripple structure can be identified, and the high-resolution TEM image in Figure 2d also attests to the evident crystalline structure in the NiCo_2_O_4_ hollow sphere. More detailed structure information was further determined by XRD and N_2_ adsorption–desorption tests.

**Figure 3:**
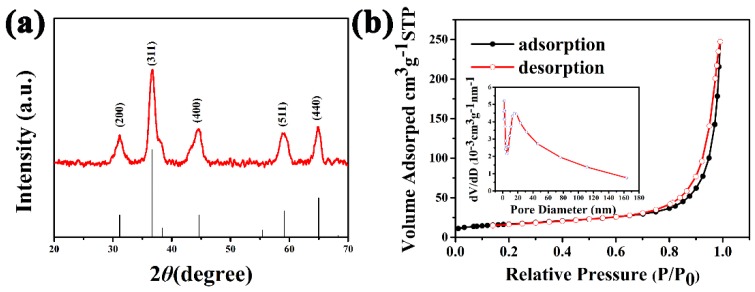
(**a**) X-ray diffraction (XRD) pattern of as-prepared NiCo_2_O_4_ hollow sphere sample; (**b**) N_2_ adsorption–desorption isotherms, and inset is the corresponding pore size distribution of the as-prepared hierarchical NiCo_2_O_4_ hollow sphere.

Figure 3a depicts the typical XRD pattern of the as-prepared materials, in which all diffraction peaks can be assigned to the spinal-structured NiCo_2_O_4_ (JCPDS No. 20-0781). The peaks at 2θ values of 31°, 36°, 45°, 59°, and 65° are corresponding to (200), (311), (400), (511), and (440) crystal faces of NiCo_2_O_4_ in cubic phase [31]. No other impurity peaks appear, indicating the high purity of the samples. The porosity of the hierarchical NiCo_2_O_4_ hollow sphere was assessed by N_2_ adsorption–desorption test as shown in Figure 3b, which gives a typical IV-type isotherm. The specific surface area estimated by Brunauer–Emmett–Teller (BET) method is 59.4 m^2^·g^−1^, and the inserted pore size distribution curve demonstrates the existence of mesopores [32]. Apparently, the porous structure of the hierarchical NiCo_2_O_4_ hollow sphere is beneficial to supply more mass transport pathways and active sites. Furthermore, the DLS result shows that the surface of the as-obtained NiCo_2_O_4_ hollow spheres is positively charged (about 3.0 mV) in water, which is conducive to the absorption of negatively charged GOx and improves the dispersibility of the particles in the solution. Additionally, the surface composition and valence state of the hierarchical NiCo_2_O_4_ hollow sphere are also vital, since the catalytic reactions mainly happened on the surface of catalysts.

**Figure 4:**
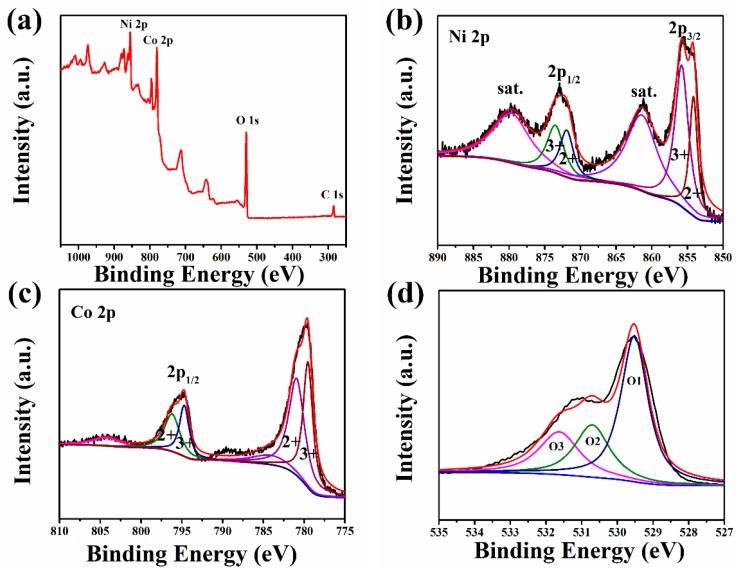
X-ray photoelectron spectroscopy (XPS) spectrum of the NiCo_2_O_4_ hollow sphere: (**a**) survey; (**b**) Ni 2p; (**c**) Co 2p; (**d**) O 1s.

XPS was applied to study the element and valance information of NiCo_2_O_4_ hollow sphere. The survey spectrum in Figure 4a demonstrates the existence of nickel, cobalt, oxygen, and slight carbon atoms. By using a Gaussian fitting, the high-resolution spectrum of Ni 2p exhibits two spin-orbit doublets (Ni^2+^ and Ni^3+^) and two shakeup satellites (Figure 4b). The peaks at 854.2 eV and 871.9 eV are attributed to Ni^2+^, and the peaks at 855.8 eV and 873.5 eV can be assigned to Ni^3+^. The Co 2p spectrum can also be fitted into two spin-orbit doublets (Co^2+^ and Co^3+^) and two shakeup satellites (Figure 4c) in the same way. These results are well in agreement with many other reports, demonstrating the Ni^2+^ and Ni^3+^, Co^2+^ and Co^3+^ inside the NiCo_2_O_4_ hollow sphere [33]. The high-resolution spectrum of O 1s exhibits three oxygen species marked as O1, O2, and O3. Based on the previous reports, O1 (with a binding energy of 529.5 eV) was a typical metal–oxygen bond, and O2 (with a binding energy of 530.7 eV) was generally attributed to defects, impurities, and some low oxygen coordination within the spinal structure [34]. As for O3 (with a binding energy of 531.6 eV), it was associated with physi- and chemisorbed water molecules [35]. The rich surface chemical composition and valence state of the NiCo_2_O_4_ hollow sphere may lead to a large number of distributional differences in local electronic cloud density, which greatly affect the catalytic activity.

### 3.2. Synthesis Mechanism

**Figure 5:**
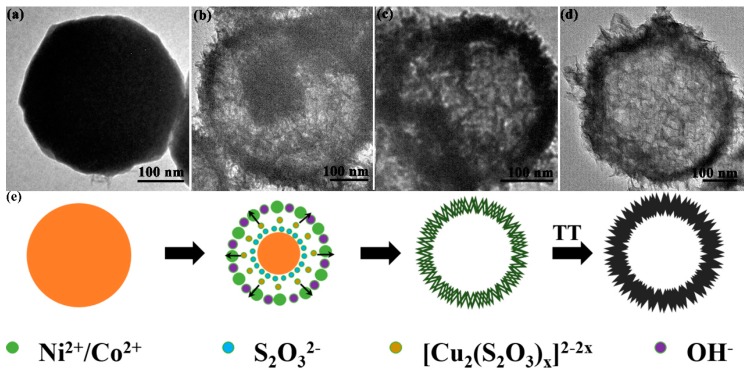
Representative TEM images of the samples at different stages: (**a**) Cu_2_O; (**b**) Cu_2_O@Ni–Co hydroxide; (**c**) Ni–Co hydroxide hollow sphere precursor; and (**d**) NiCo_2_O_4_ hollow sphere. (**e**) The corresponding schematic illustration of the formation process for hierarchical NiCo_2_O_4_ hollow sphere. Abbreviations: TT, thermal treatment.

Based on the above discussion, the well-designed “CEP” process has been successfully applied to the synthesis of the hierarchical porous NiCo_2_O_4_ hollow sphere. Figure 5a–d display the evolution in morphology and structure, from Cu_2_O solid sphere to the final hierarchical NiCo_2_O_4_ hollow sphere. Figure 5e displays the corresponding schematic illustration of the formation process. As is well known, according to the HSAB (hard and soft acids and bases) theory, Cu^+^ has much stronger interaction with S_2_O_3_^2−^ (soft–soft interaction) than that of O^2−^ (soft–hard interaction). Thus, the presence of S_2_O_3_^2−^ would lead to the elimination of the Cu_2_O template and an increased local OH^−^ concentration on the etching interface. Besides, accompanied by the hydrolyzation of S_2_O_3_^2−^ and coprecipitation of metal ions with OH^−^, Ni–Co hydroxide units are preferential to nucleation and growth at the etching interface to form the Ni–Co hydroxide hollow sphere (Figure 5b). It is noteworthy that both the template elimination and duplication processes can be accurately controlled by simply adjusting the ratio of water to ethanol.

### 3.3. Peroxidase-Like Activity of the Hierarchical NiCo_2_O_4_ Hollow Sphere

**Figure 6:**
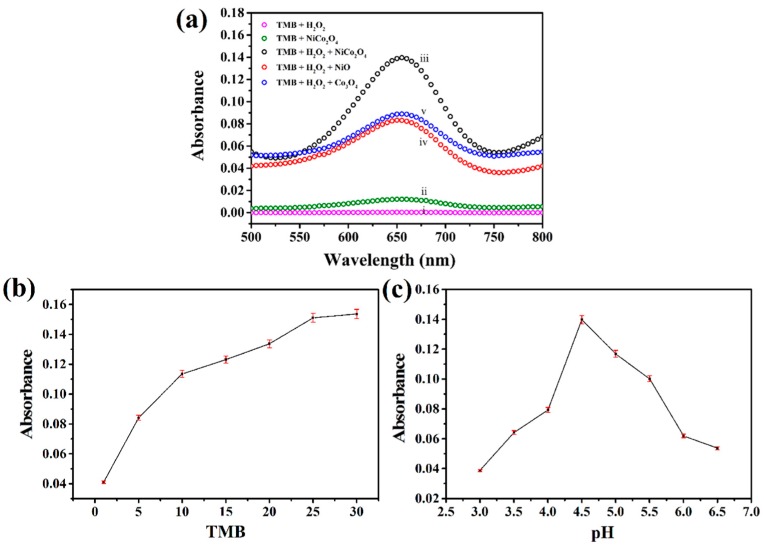
(**a**) Photograph and absorption spectra of colorimetric reactions under different conditions: (i) 3,3,5,5-tetramethylbenzidine (TMB) + H_2_O_2_; (ii) TMB + NiCo_2_O_4_; (iii) TMB + H_2_O_2_ + NiCo_2_O_4_; (iv) TMB + H_2_O_2_ + NiO; (v) TMB + H_2_O_2_ + Co_3_O_4_. TMB concentration- (**b**) and pH- (**c**) dependent peroxidase-like activity of hierarchical NiCo_2_O_4_ hollow sphere. Experimental conditions: 5 μL of 1 mg·mL^−1^ enzyme mimetic dispersions were incubated with 3 mL of pre-prepared sodium citrate buffer solution (0.1 M, pH 4.5) with the presence of H_2_O_2_ (0.02 M) and TMB (0.08 mM).

To investigate the peroxidase-like activity of the hierarchical NiCo_2_O_4_ hollow sphere, the catalysis of the peroxidase substrate TMB was performed in presence or absence of H_2_O_2_. Additionally, the peroxidase-like activities of NiO and Co_3_O_4_ hollow spheres synthesized by the same method were also studied for comparison. Thus, five controlled experiments were carried out: (i) TMB + H_2_O_2_; (ii) TMB + NiCo_2_O_4_; (iii) TMB + H_2_O_2_ + NiCo_2_O_4_; (iv) TMB + H_2_O_2_ + NiO; (v) TMB + H_2_O_2_ + Co_3_O_4_. As shown in Figure 6a, only with the coexistence of H_2_O_2_ and NiCo_2_O_4_ catalyst could beget an obvious absorption peak at 652 nm (iii) due to the oxidation of TMB, which indicates a peroxidase-like activity. As in previous reported work, both of the as-prepared NiO (iv) and Co_3_O_4_ (v) catalysts exhibited peroxidase-like activity in this research. However, the corresponding absorption spectra reveals that the hierarchical NiCo_2_O_4_ hollow sphere obviously possesses a stronger absorption peak than that of NiO and Co_3_O_4_ under the parallel condition, suggesting an enhanced peroxidase-like activity. The possible reaction mechanism is different from that of Fe-based catalysts, which are mainly conducted by the “Fenton” reaction. During this process, TMB molecules are absorbed on the surface of the hierarchical NiCo_2_O_4_ hollow sphere, and can donate lone-pair electrons from the amino groups to the catalyst [8]. That results in an increase in electron density and mobility in the NiCo_2_O_4_ hollow sphere, which would facilitate the electron transfer from the NiCo_2_O_4_ hollow sphere to H_2_O_2_ to accelerate the oxidation rate of TMB by H_2_O_2_ [9]. Furthermore, the enhanced peroxidase-like activity of the NiCo_2_O_4_ catalyst should be attributed to the diversified distribution of surface electron clouds and the hierarchical porous hollow structure that could provide ample active sites for catalytic reaction. The peroxidase-like activity of the hierarchical NiCo_2_O_4_ hollow sphere is also dependent on the TMB concentration and pH environment. As shown in Figure 6b, the absorption intensity at 652 nm was increased with the increasing of TMB, but no obvious increase was observed when the concentration was greater than 0.08 mM (25 μL), suggesting an optimized TMB concentration. Additionally, the pH-dependent peroxidase-like activity in Figure 6c indicates that the optimal pH should be 4.5.

### 3.4. Colorimetric Detection of H_2_O_2_

**Figure 7:**
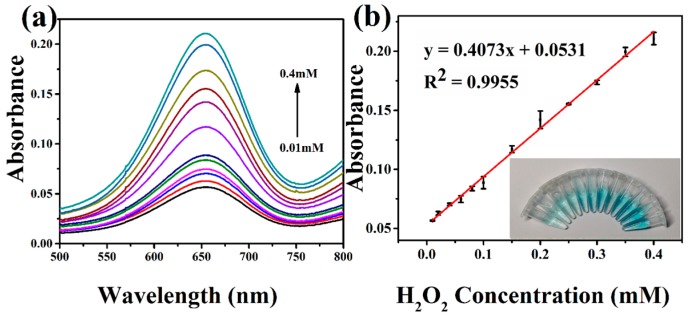
(**a**) Absorption spectra of the hierarchical NiCo_2_O_4_ hollow sphere with various concentrations of H_2_O_2_ (0.01–0.4 mM). (**b**) The corresponding linear calibration curve. The inset shows the photograph of color change for various concentrations.

Based on the above peroxidase-like activity investigation of the hierarchical NiCo_2_O_4_ hollow sphere, a colorimetric method was developed for detection of H_2_O_2_. In Figure 7a, the absorbance at 652 nm for oxidized TMB increases with the concentration of H_2_O_2_. Figure 7b shows the corresponding linear calibration curve and photograph of color change. The linear range is from 0.01 mM to 0.4 mM, and the detection limit is calculated to be 0.21 μM. Notably, the concentration-dependent color change could offer a handy way for detection of H_2_O_2_ by naked eye.

### 3.5. Colorimetric Detection of Glucose

**Figure 8:**
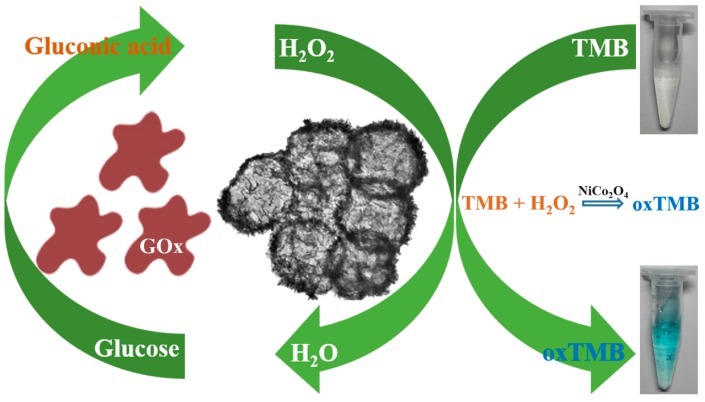
Scheme illustration for colorimetric detection of glucose using the hierarchical NiCo_2_O_4_ hollow sphere.

**Figure 9:**
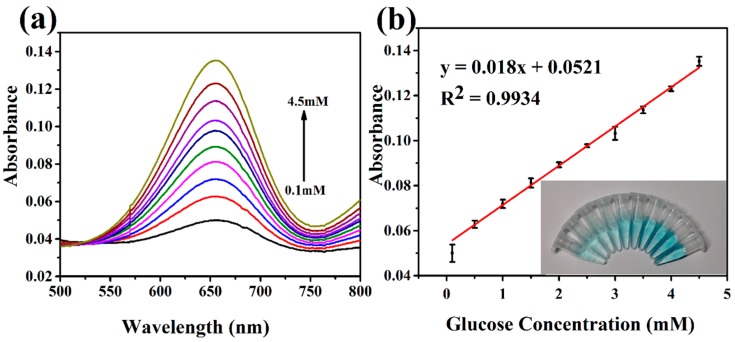
(**a**) Absorption spectra of the hierarchical NiCo_2_O_4_ hollow sphere with various concentrations of glucose (0.1–4.5 mM). (**b**) The corresponding linear calibration curve. The inset shows the photograph of color change for various concentrations.

The concentration of blood glucose is an important physiological indicator for the human body. The detection of glucose is meaningful for clinical diagnosis and treatment of disease. H_2_O_2_ is one of the main intermediate products of glucose oxidase-catalyzed reaction. To take full advantage of the peroxidase-like activity of the hierarchical NiCo_2_O_4_ hollow sphere, the colorimetric detection of glucose was also developed, and the reaction mechanism is illustrated in Figure 8. As shown in Figure 9a, the absorbance at 652 nm for oxidized TMB increases with the concentration of H_2_O_2_. The corresponding linear calibration curve and photograph of color change in Figure 9b shows that the linear range is from 0.1 mM to 4.5 mM with a detection limit of 5.31 μM. It can be seen from Table 1 that the proposed catalyst performs satisfactory combination properties toward glucose sensing compared with most non-Pt-based catalysts. Even though the present NiCo_2_O_4_-based sensor exhibits slightly lower sensing performance to that of a Pt-based sensor, but it is also a low-cost and effective detection method. Moreover, the concentrations of blood glucose in healthy and diabetic persons is generally in the range of 3–8 mM and 9–40 mM, respectively [36]. Therefore, the proposed method is applicable to glucose analysis in real serum by simple dilution.

### 3.6. Selectivity, Reproducibility, and Applicability

**Figure 10:**
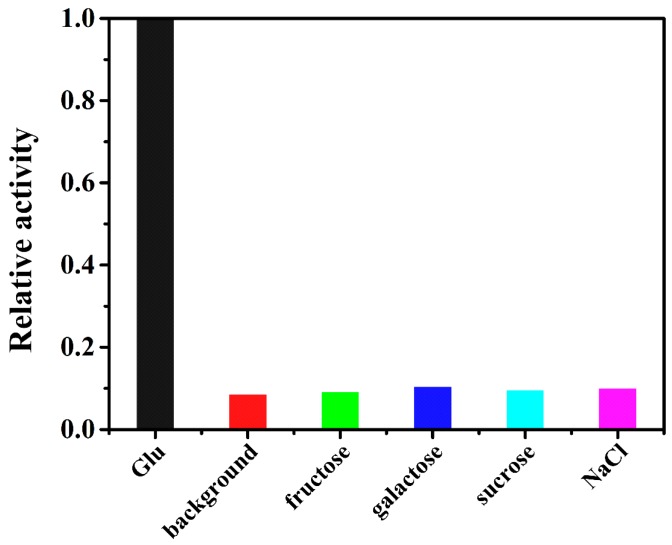
Selectivity of the proposed sensor for glucose detection was assessed by measuring the absorption intensity at 652 nm. The concentrations for fructose, galactose, sucrose, and NaCl are 20 mM, respectively.

The selectivity, reproducibility, and applicability of the proposed sensor for glucose detection were investigated. As shown in Figure 10, the presence of glucose apparently produced much stronger absorption intensity and color change than that of other analytes, indicating a good selectivity to glucose of this biosensor. The reproducibility was evaluated by three consecutive detections, resulting in an acceptable relative standard deviation (RSD) of 3.24%. For applicability, the sensor was applied to detect the human serum sample: the detected glucose concentration was 4.2 mM with an acceptable RSD of 4.5%, compared to the concentration of 4.4 mM determined by the hospital.

## 4. Conclusions

In summary, the hierarchical NiCo_2_O_4_ hollow sphere was successfully synthesized by a “coordinating etching and precipitating” process and exhibited intrinsic peroxidase-like activity. The experimental results revealed that the hierarchical NiCo_2_O_4_ hollow sphere possessed superior peroxidase-like activity to that of single NiO or Co_3_O_4_. For use as a peroxidase mimic, the proposed sensing platform showed a commendable response to H_2_O_2_ with a linear range from 10 μM to 400 μM and a detection limit of 0.21 μM. More importantly, combined with GOx, the developed glucose-sensing platform exhibited high selectivity, superb reproducibility, satisfactory applicability, wide linear range from 0.1 mM to 4.5 mM, and low detection limit of 5.31 μM. Additionally, the concentration-dependent color change could offer a handy way for detection of H_2_O_2_ and glucose by naked eye. Therefore, the hierarchical NiCo_2_O_4_ hollow sphere would be a promising candidate for colorimetric detection of H_2_O_2_ and glucose.

## Figures and Tables

**Table 1:** Comparison of the linear ranges and the detection limits with other methods for the detection of glucose.

Catalyst	Linear Range (μM)	**Detection Limit (**μ**M)**	**Reference**
NiCo_2_O_4_	100–4500	5.31	This work
CuO	100–8000	-	[37]
V_2_O_5_	10–2000	10	[3]
Au nanoparticles	30–1000	30	[38]
FeCPNPs	2–20	1	[39]
H_2_TCPP–Co_3_O_4_ nanocomposites	1–10	0.86	[40]
Zn^2+^/AMP nanofiber	-	0.6	[41]
CeO_2_ nanoparticle	-	8.9	[42]
Pt–DNA complexes	0.1–1000	0.1	[43]

FeCPNPs: Fe (III)-based coordination polymer nanoparticles. H_2_TCPP: 5,10,15,20-Tetrakis(4-carboxyl pheyl)-porphyrin. AMP: Adenosine 5′-monophosphate.
